# Local Anesthetic Systemic Toxicity After Local Infiltration Analgesia Following a Total Knee Arthroplasty

**DOI:** 10.7759/cureus.26224

**Published:** 2022-06-23

**Authors:** Randolph Kapenda Lungonyonyi, Pauline Bleuze, Jean-Paul Boutière

**Affiliations:** 1 Anesthesiology and Reanimation, Université Catholique de Louvain, Namur, BEL; 2 Anesthesiology and Reanimation, Centre Hospitalier Intercommunal Nord-Ardenne Charleville-Mézières, Charleville-Mézières, FRA

**Keywords:** pain management, total knee arthroplasty, local infiltration analgesia, local anesthesic systemic toxicity, ropivacaine

## Abstract

Multimodal analgesia is nowadays a recognized method to reduce postoperative pain. Infiltration of the surgical site with local anesthetic is one of the methods widely used for its effectiveness and its lower risk of toxicity, particularly in orthopedic surgery. We present here a case of local anesthetic systemic toxicity (LAST) that occurred on two different occasions to the same patient after total knee replacement surgeries. The clinical presentation of the syndrome was not typical for this 74-year-old patient who presented with respiratory distress associated with impaired consciousness. After the elimination of the most common diagnoses, it was finally the administration of Intralipid that followed with clinical improvement.

## Introduction

Local infiltration analgesia (LIA) is commonly used for the prevention of postoperative pain in knee surgery. Even though high doses of local anesthetics are used, there are few case reports of systemic toxicity in the literature regarding this technique [1,2]. We present here a case of systemic toxicity of late-onset, which happened on two separate occasions to the same patient after infiltration of the knee with 200mL of Ropivacaine 2mg/mL in the context of total knee replacement surgery.

## Case presentation

A 74-year-old woman presented for total knee arthroplasty. Her American Society of Anesthesiology (ASA) was 2. She had previously undergone bilateral hip replacement surgery and a narrow lumbar canal cure, all of which were without complications. Her medical history included arterial hypertension, and morbid obesity (BMI 38.28kg/m² with a height of 160cm and a weight of 98kg). Her preoperative biological workup did not reveal any abnormalities, with preserved renal function, normal liver function, and no evidence of malnutrition.

The procedure was performed under general anesthesia. The patient had standard monitoring (electrocardiogram, SpO_2_, noninvasive blood pressure measurement, neuromuscular monitoring). Induction was done with sufentanil (20µg), propofol (200mg), ketamine (20mg), and rocuronium (100mg). Anesthesia was then maintained with sevoflurane. Anesthesia and surgery were uneventful.

For postoperative analgesia, nonsteroidal anti-inflammatory drug (ketoprofen 100mg) and paracetamol (1g) were given to the patient and the surgeon performed a local anesthetic infiltration, using a standardized institutional including ropivacaine 2mg/mL with epinephrine, for a total of 400mg of ropivacaine and 1mg of epinephrine, associated with corticosteroids (60mg of methylprednisolone). Injections were made intra and periarticular and in the integuments.

Sugammadex (400mg) was administrated and the neuromuscular monitoring showed no residual curare effect. At the end of the operation, the patient was transferred to the recovery room where she was extubated without complication. She was discharged after two hours of monitoring and carried to the surgical floor. She had no painful manifestation, after having received a total of 7mg of morphine and she needed oxygen therapy with a nasal cannula (3L/min).

The patient was then readmitted in an emergency to the recovery room just 1 hour after having been discharged for respiratory distress with desaturation, with a SpO_2_ level of 64% and a neurological picture of confusion and altered consciousness (Glasgow Coma Scale, GCS 8-9). The patient received aerosol therapy (ipratropium, terbutaline, epinephrine), associated with a naloxone test to eliminate a residual morphine impregnation. In the absence of clinical improvement, the patient was intubated and transferred to the intensive care unit.

During her stay in the intensive care unit (ICU), corticosteroid (methylprednisolone 1mg/kg) was started because of a suspicion of epiglottitis during laryngoscopy. But an ENT endoscopy finally ruled out this diagnosis. A chest x-ray was performed, which did not show atelectasis or a clear radiological image of pneumonia and the test came back negative for SARS-CoV-2. The patient improved rapidly on the respiratory level allowing extubation. No cardiac, neurological, or renal complications were encountered, and no clear reason, at that moment, could be found for that respiratory distress.

The patient returned eight months later for contralateral knee arthroplasty. Initially scheduled under spinal anesthesia, the procedure was finally performed under general anesthesia after failed attempts. The anesthesia and surgery were again performed without any particularity and the patient was again infiltrated following the same protocol (ropivacaine 2mg/mL with epinephrine, for a total of 400mg of ropivacaine and 1mg of epinephrine, associated with corticosteroids, 60mg of methylprednisolone). The patient was then taken to the recovery room where she was extubated under excellent conditions. A quarter of an hour after extubation, she presented with the same symptomatology she had months earlier with dyspnea (decreased thoracic ampliation, desaturation down to SpO_2_ of 89%) and consciousness disorders (GCS 8-9). She again received aerosol therapy (adrenaline), non-invasive ventilation, and intravenous corticosteroid injection (methylprednisolone 120mg). After the failure of respiratory symptomatic treatment, a suspicion of local anesthetics systemic toxicity (LAST) was raised. Intralipid treatment (1mL/kg of Intralipid 20% for a total of 200mL) was therefore introduced, allowing the patient to rapidly recover her respiratory and neurological functions. She was able to return to the surgical floor after two hours in the recovery room without any support. The rest of the evening and night passed without any other event. She did not present with any complications during her hospitalization and left the orthopedic department after seven days. The patient underwent a complete allergological workup, with testing for common pneumallergens as well as for the products used during the procedure, including ropivacaine, which proved negative.

## Discussion

While we were unable to determine plasma ropivacaine concentrations, the elimination of other diagnoses and resolution of symptoms after intralipid administration support the diagnosis of LAST. LAST is a rarely described complication in the literature, with a 2018 review listing 47 cases over the period from 2014 to 2016, 17% of which were after an infiltration [1]. However, it should remain feared and not forgotten [3]. LAST most commonly presents with central nervous system changes [1,4]. Early signs and symptoms include agitation, confusion, dizziness, drowsiness, dysphoria, auditory changes, tinnitus, perioral numbness, metallic taste, and dysarthria. But the early or prodromal symptoms of LAST may not be recognized in the anesthetized patient. In this case, absorption may have been relatively slow or delayed because of limited local vascularization and the use of a tourniquet.

Concerns can be raised about doses of ropivacaine used that exceed the recommended maximum dose of 3-4mg/kg, as the total amount of local anesthetics injected is one of the major risk factors for LAST [5,6], but although the doses injected during LIA are very high, they are not normally related to toxic free plasma doses in the literature [2,7].

Other risk factors for toxicity have been demonstrated, including liver disorders, hypoproteinemia, albumin deficiency, and renal failure with uremia [5,6], but these were ruled out in this case. Further investigations should be conducted regarding possible ⍺-acid glycoprotein deficiency, but the main risk factor that can be retained is her advanced age [5]. In the management of frail patients, especially the elderly, with an increased risk of systemic toxicity, it would be of interest for clinicians to administer the lowest effective dose and to pay special and prolonged attention and vigilance in the postoperative period [6].

## Conclusions

Although intra-articular infiltration has proven to be effective without increasing the risk of LAST compared to femoral block, its risks should not be overlooked or underestimated. The patients most at risk of LAST, in particular elderly patients and women, should certainly benefit from lower total doses of local anesthetics and increased monitoring time in the recovery room.
